# Supplementary material for: ChineseEEG: A Chinese Linguistic Corpora EEG Dataset for Semantic Alignment and Neural Decoding
Source: Sci Data. 2024 May 29;11:550. doi: 10.1038/s41597-024-03398-7 (PMC11137001; doi:10.1038/s41597-024-03398-7)
Supplement: Supplementary file 1 — Human data submission checklist [file 41597_2024_3398_MOESM1_ESM.docx]

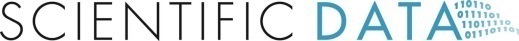
Human Data Submission Checklist

Authors submitting manuscripts to *Scientific Data* must make any described datasets available to editors and referees, in a secure and confidential manner. For datasets that require special controls to ensure ethical use, a mechanism must be provided by which our referees may access the data without revealing their identities to study authors or their institutions. If you are submitting a dataset that will require special controls to facilitate referee access, we ask that you complete and upload this form as part of your submission process.

## I – Human research participants

##### Ethical compliance

Confirm that you have complied with all relevant ethical regulations and that a statement affirming this is included in the manuscript.

##### Ethics committee

Confirm that the manuscript states the name(s) of the board and/or institution that:

Approved the study protocol -OR-  Provided guidelines for study procedures (if protocol approval is not required)

##### Informed consent

Confirm that informed consent was obtained from all participants.

##### Identifiable images

For publication of identifiable images of research participants, included in the manuscript or associated dataset, confirm that consent to publish was obtained and is noted in the Methods.

Yes  No identifiable images of human research participants

## II – Clinical studies

Policy information about [clinical studies](http://www.nature.com/authors/policies/availability.html#clinical)

##### Clinical trial registration

Confirm that you have provided the trial registration number from [ClinicalTrials.gov](https://clinicaltrials.gov/) or an equivalent agency in the manuscript.

##### Phase 2 and 3 randomized controlled trials

Confirm that you have provided the [CONSORT checklist](http://www.consort-statement.org/consort-statement/checklist) with your submission.

Yes  No  Not a phase 2/3 randomized controlled trial

##### Tumour marker prognostic studies

Did you follow the [REMARK reporting guidelines](http://www.nature.com/nrclinonc/journal/v2/n8/full/ncponc0252.html)?

Yes  No  Not a tumour marker prognostic study

## III – Referee data access

Please select one of the options below:

My data do not require special access controls, and can be accessed directly from a repository approved by *Scientific Data* using the information provided with my submission.

My data are hosted at UKDA ReShare, and will be available to referees via their standard policies for reviewer access (<http://reshare.ukdataservice.ac.uk/reshare-review-procedures/>).

My data are hosted at Synapse, and require only a valid Synapse account to gain access.

My data are hosted at Synapse, and access will be controlled by a separate Referee Data Access Agreement. (Please provide a copy of a Referee Data Access Agreement with your submission. Examples are available upon request.)

Other: please outline briefly the process by which referees may gain access to the data in the box below. If you do not know how to complete this box, please contact [scientificdata@nature.com](mailto:scientificdata@nature.com):

Click here to enter text.

We understand that for some datasets anonymous peer-review may not be possible to arrange. We regret, however, that we cannot consider such datasets at the journal.

## IV – Data Access After Publication

If access to the data after publication will be controlled by a data use agreement (DUA), please provide a link to the DUA (if available on the web) or upload a copy as a supplementary file.

na

Does the DUA require other researchers to list the data generators as authors on subsequent works or require collaboration, as a condition of data access?

Yes  No

Please note that datasets with co-authorship or collaboration requirements will only be considered at the journal when our Editorial Board feels the conditions are justified and would not prevent critical or competitive reuse. See <https://www.nature.com/articles/sdata2017133>.

By submitting this form to the journal you confirm that your data release plan is consistent with consent obtained in this case, and complies with all ethical and legal requirements that may apply.

I certify that all the above information is complete and correct.

Feb 9, 2024

Haiyan WU

Typed signature Date
